# Influence of Intestinal Barrier on Alleviating an Increase in Blood Pressure by Sodium Alginate Intake in 2-Kidney, 1-Clip Renovascular Hypertensive Rats

**DOI:** 10.3390/md21060324

**Published:** 2023-05-26

**Authors:** Saki Maruyama, Yukiko Segawa, Ayaka Harui, Kanae Yamamoto, Hiroko Hashimoto, Tomoko Osera, Nobutaka Kurihara

**Affiliations:** 1 Hygiene and Preventive Medicine, Graduate School of Home Economics, Kobe Women’s University, 2-1 Higashisuma-Aoyama, Suma, Kobe 654-8585, Japan; s-maruyama@suma.kobe-wu.ac.jp (S.M.); segawa@osaka-seikei.ac.jp (Y.S.); hashimoto-hi@osaka-seikei.ac.jp (H.H.); osera083@toyo.jp (T.O.); 2 Faculty of Cookery and Confectionery, Osaka Seikei College, 10-62 Aikawa, Higashiyodogawa, Osaka 533-0007, Japan; 3 Faculty of Nutrition, Osaka Seikei College, 10-62 Aikawa, Higashiyodogawa, Osaka 533-0007, Japan; 4 Department of Nutrition and Health Sciences, Toyo University, 1-1-1 Izumino, Ora-gun, Itakura-machi 374-0193, Gunma, Japan

**Keywords:** sodium alginate, prevention of hypertension, renovascular hypertension, gut barrier

## Abstract

Sodium alginate (SALG) is a substance derived from brown seaweed that has been shown to reduce blood pressure (BP). However, its effects on renovascular hypertension caused by 2-kidney, 1-clip (2K1C) are not yet clear. Previous research suggests that hypertensive rats have increased intestinal permeability, and that SALG improves the gut barrier in inflammatory bowel disease mouse models. Therefore, the goal of this study was to determine whether the antihypertensive effects of SALG involve the intestinal barrier in 2K1C rats. Rats were fed either a 1.0% SALG diet or a control diet for six weeks after being subjected to 2K1C surgery or a sham operation. The systolic BP was measured weekly, and the mean arterial BP was measured at the end of the study. Intestinal samples were taken for analysis, and plasma lipopolysaccharide (LPS) levels were measured. The results showed that BP in 2K1C rats was significantly higher than in SHAM rats when fed CTL, but not when fed SALG. The gut barrier in 2K1C rats was improved by SALG intake. Plasma LPS levels also differed depending on the animal model and diet. In conclusion, dietary SALG may alleviate 2K1C renovascular hypertension by altering the gut barrier.

## 1. Introduction

Hypertension is a strong risk factor for total mortality and cardiovascular disease [1]; therefore, preventing hypertension is crucial. Diet is one of the many approaches to controlling hypertension. We demonstrated that the blood pressure (BP) of 2-kidney, 1-clip (2K1C) renovascular hypertensive rats fed a diet containing *Saccharina japonica* significantly decreased compared to that of 2K1C rats fed a control diet in our previous study [2]. *Saccharina japonica* contains sodium alginate, which has been reported to decrease BP in other hypertensive rats, as shown below. Therefore, we focused on the antihypertensive effects of sodium alginate intake in 2K1C rats.

Alginate is a naturally occurring polyuronic acid composed of two conformational isomer residues, namely, β-D-mannuronic acid (M) and α-L-guluronic acid (G), linked by β-1,4-glycosidic bonds. The variety of biological activities in alginate include antioxidant activities [3], mucosal protective effects [4,5], antiobesity [6], antihypertension [7,8,9,10,11], and gut microbiota alteration [8,12]. Among others, BP increase induced by an intake of 1.0% NaCl drinking water was attenuated by a 1.0% sodium alginate diet consumed for 20 days in Wistar rats [7]. Additionally, the administration of potassium alginate oligosaccharide to spontaneously hypertensive rats (SHR) for 21 days and 6 weeks and of deoxycorticosterone acetate (DOCA)-salt rats for 30 days inhibited BP increase in a dose-dependent manner [8,9]. Furthermore, according to previous reports, the intake of 4.0% or 8.0% sodium alginate oligosaccharide diet for 7 weeks attenuated BP increase in a dose-dependent manner [10]. A study demonstrated that in Dahl-salt rats fed a high-salt diet, the use of a continuous subcutaneous osmotic micropump of sodium alginate oligosaccharide administration (60 mg/day) almost eliminated hypertension [13].

A leaky intestinal barrier has been previously reported, characterized by increased permeability, combined with altered intestinal tight junction proteins including an increase in serum or plasma lipopolysaccharide (LPS) levels in SHR, angiotensin II-induced hypertensive rats and DOCA-salt hypertensive rats [14,15,16,17,18,19]. LPS, the main components of the outer membrane of Gram-negative bacteria, is an endotoxin and affects the composition of the intestinal flora and destroys the gut mucosal barrier. As a result, it greatly increases the reproduction and translocation of harmful bacteria, causing metabolic endotoxemia [20]. LPS activates the endothelial toll-like receptor 4, and induces endothelial dysfunction via the nicotinamide adenine dinucleotide phosphate oxidase/reactive oxygen species/endothelial nitric oxide synthase pathway and vascular inflammation via the mitogen-activated protein kinase and nuclear factor kappa B pathways [21]. A previous study reported that captopril, one of the angiotensin-converting enzymes, alleviated hypertension in SHR and improved the gut barrier [14,22]. It is also reported that sodium alginate reversed intestinal mucosal barrier function in dextran sulfate sodium (DSS)-induced and cyclophosphamide-induced immunosuppressed BALB/c mice [23,24]. Therefore, we hypothesized that the effect of the gut barrier by alginate is involved in the mechanism of the antihypertensive effect of sodium alginate in 2K1C renovascular hypertensive rats. However, to the best of our knowledge, there has been no reported study investigating the mechanism of the antihypertensive effect of sodium alginate from the perspective of the gut barrier.

In this study, we explored the effects of sodium alginate intake for 6 weeks on BP in 2K1C rats. Then, we investigated whether the gut barrier participates in the inhibitory effect of sodium alginate on hypertension in the 2K1C model by measuring gut morphology, intestinal tight junction proteins, and plasma LPS levels. Our finding indicates that sodium alginate intake may attenuate hypertension in 2K1C rats by altering the gut barrier.

## 2. Results

### 2.1. Effects of Dietary Sodium Alginate Intake on BP in SHAM and 2K1C Rats

**Figure 1:**
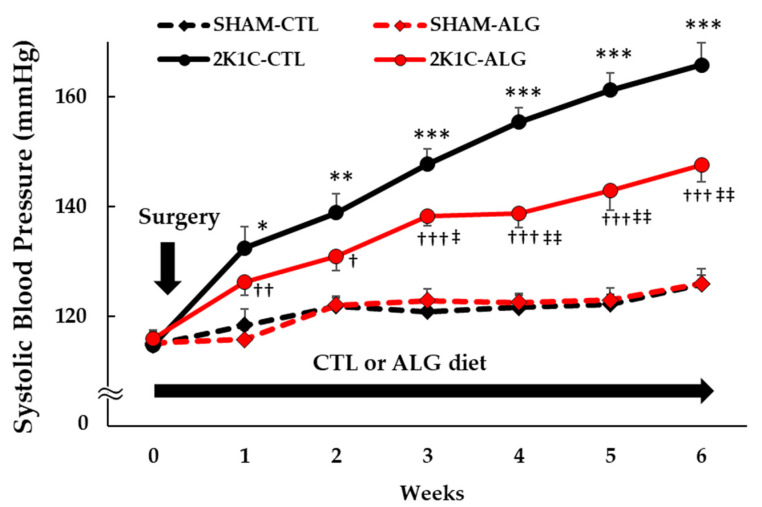
Systolic blood pressure obtained using a tail-cuff method in SHAM or 2K1C rats fed CTL or ALG for 6 weeks. Values are expressed as mean ± SE, *n* = 6. Three-way ANOVA: *p* < 0.001 for time, animal (SHAM vs. 2K1C), diet (CTL vs. ALG), time x animal, animal x diet, and *p* < 0.05 for time x animal x diet. * *p* < 0.05, ** *p* < 0.01 and *** *p* < 0.001 vs. SHAM-CTL, † *p* < 0.05, †† *p* < 0.01 and ††† *p* < 0.001 vs. SHAM-ALG, ‡ *p* < 0.05 and ‡‡ *p* < 0.01 vs. 2K1C-CTL. Abbreviations: SHAM, sham-operated control rats; 2K1C, 2-kidney, 1-clip hypertensive rats; CTL, a control diet; ALG, a diet with sodium alginate; SE, standard error; ANOVA, analysis of variance.

**Figure 2:**
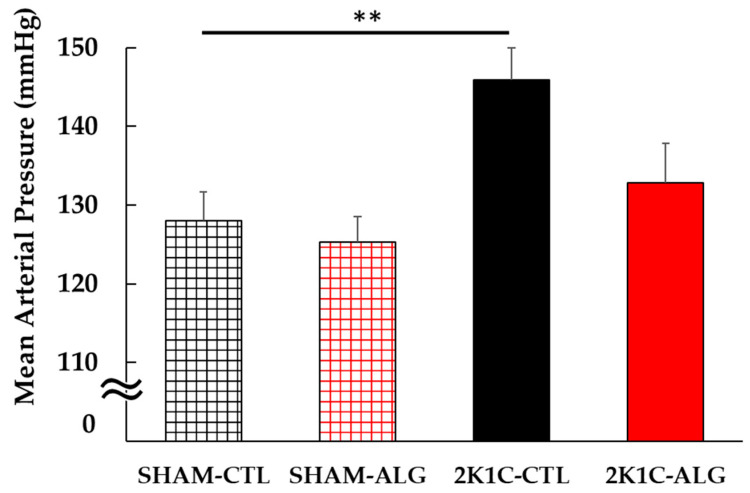
Mean arterial blood pressure in SHAM or 2K1C rats fed CTL or ALG under anesthesia at the end of the protocol. Values are mean ± SE, *n* = 5–6. Two-way ANOVA showed *p* < 0.05 for animal (SHAM vs. 2K1C). ** *p* < 0.01. Abbreviations: SHAM, sham-operated control rats; 2K1C, 2-kidney, 1-clip hypertensive rats; CTL, a control diet; ALG, a diet with sodium alginate; SE, standard error; ANOVA, analysis of variance.

The Systolic BP (SBP) was significantly higher in the 2K1C rats fed a control diet (2K1C-CTL) than in the sham-operated (SHAM) rats fed a control diet (SHAM-CTL) in the last week (166 ± 4 vs. 126 ± 2 mmHg, *p* < 0.001; Figure 1). Compared with 2K1C-CTL, a significant reduction in SBP was observed in the 2K1C rats fed with a 1.0% sodium alginate diet (2K1C-ALG) in the last week (148 ± 3 mmHg, *p* < 0.01). However, no significant difference in SBP was observed between SHAM-CTL and SHAM rats fed an ALG diet (SHAM-ALG, 126 ± 3 mmHg). The mean arterial BP (MAP) was significantly higher in 2K1C-CTL than in SHAM-CTL (146 ± 4 vs. 128 ± 4 mmHg, *p* < 0.01; Figure 2). No significant difference in the MAP was observed between SHAM-CTL and SHAM-ALG (125 ± 3 mmHg). Compared with 2K1C-CTL, a marginal reduction in MAP was observed in 2K1C-ALG (133 ± 5 mmHg), although there is no significant difference. These findings indicate that dietary sodium alginate intake attenuates increased BP in the hypertensive animal models, 2K1C rats, but not in the normotensive controls, SHAM rats.

### 2.2. Effects of Dietary Sodium Alginate Intake on Gut Morphology in SHAM and 2K1C Rats

**Figure 3:**
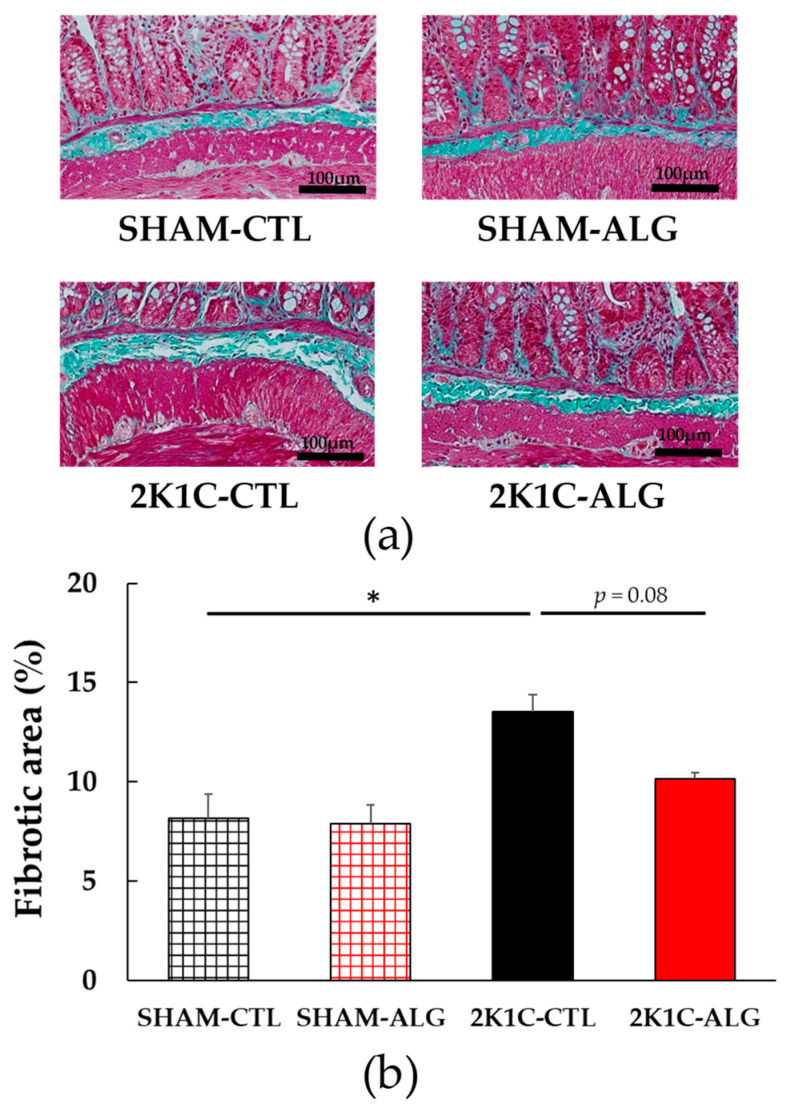
Fibrotic area in the ileum in SHAM or 2K1C rats fed CTL or ALG for 6 weeks. (**a**) Representative micrographs of Masson’s trichrome staining and (**b**) Quantitative Fibrotic area of Masson’s trichrome staining. Values are expressed as mean ± SE, *n* = 6. Two-way ANOVA: *p* < 0.01 for animal (SHAM vs. 2K1C) and *p* < 0.05 for diet (CTL vs. ALG). * *p* < 0.05. Abbreviations: SHAM, sham-operated control rats; 2K1C, 2-kidney, 1-clip hypertensive rats; CTL, a control diet; ALG, a diet with sodium alginate; SE, standard error; ANOVA, analysis of variance.

**Figure 4:**
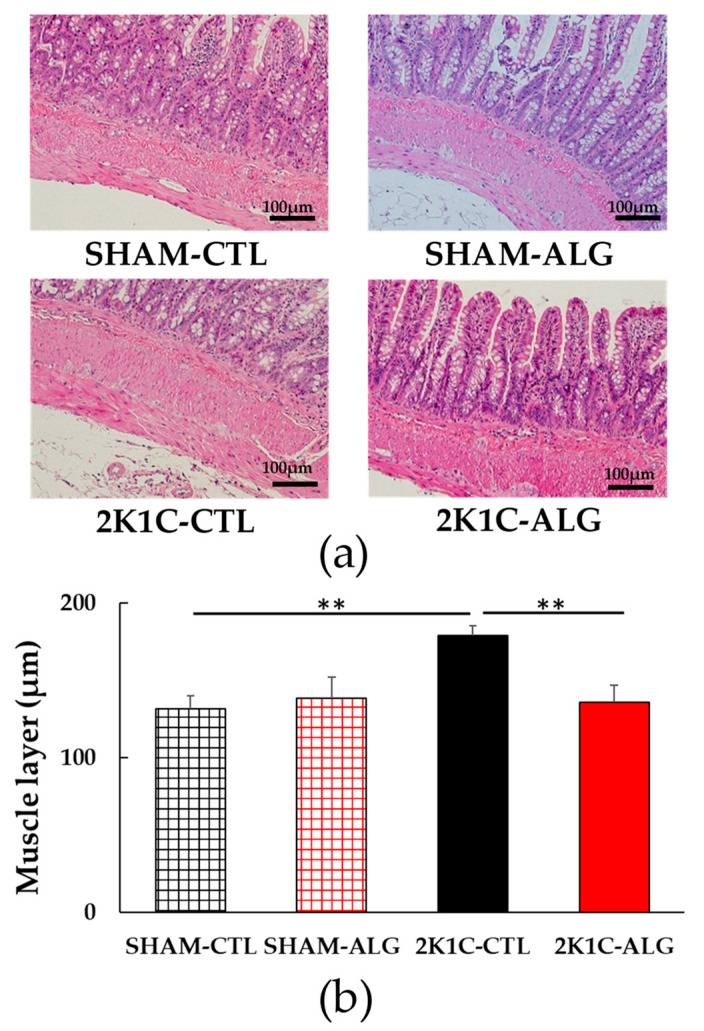
Muscle layer in the ileum in SHAM or 2K1C rats fed CTL or ALG for 6 weeks. (**a**) Representative micrographs of H&E staining and (**b**) Quantitative Muscle layer of H&E staining. Values are expressed as mean ± SE, *n* = 6. Two-way ANOVA: *p* < 0.05 for animal (SHAM vs. 2K1C) and animal x diet (CTL vs. ALG). ** *p* < 0.01. Abbreviations: SHAM, sham-operated control rats; 2K1C, 2-kidney, 1-clip hypertensive rats; CTL, a control diet; ALG, a diet with sodium alginate; SE, standard error; ANOVA, analysis of variance.

**Figure 5:**
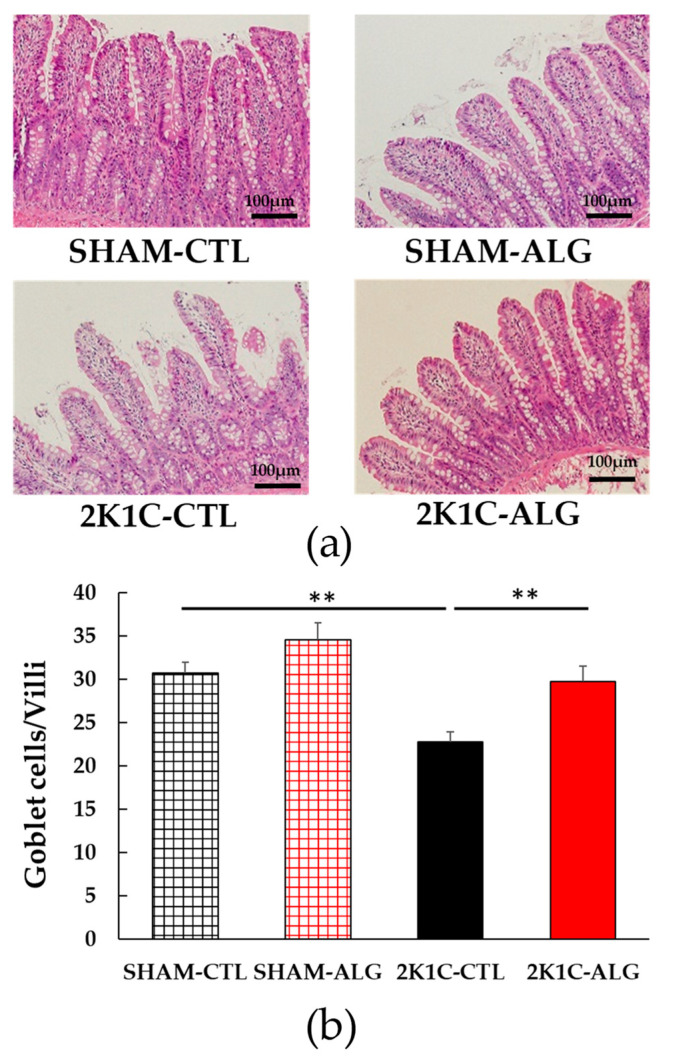
The number of goblet cells/villi in the ileum in SHAM or 2K1C rats fed CTL or ALG for 6 weeks. (**a**) Representative micrographs of H&E staining and (**b**) Quantitative the number of goblet cells/villi of H&E staining. Values are expressed as mean ± SE, *n* = 6. Values are expressed as mean ± SE, *n* = 6. Two-way ANOVA: *p* < 0.01 for animal (SHAM vs. 2K1C) and diet (CTL vs. ALG). ** *p* < 0.01. Abbreviations: SHAM, sham-operated control rats; 2K1C, 2-kidney, 1-clip hypertensive rats; CTL, a control diet; ALG, a diet with sodium alginate; SE, standard error; ANOVA, analysis of variance.

**Figure 6:**
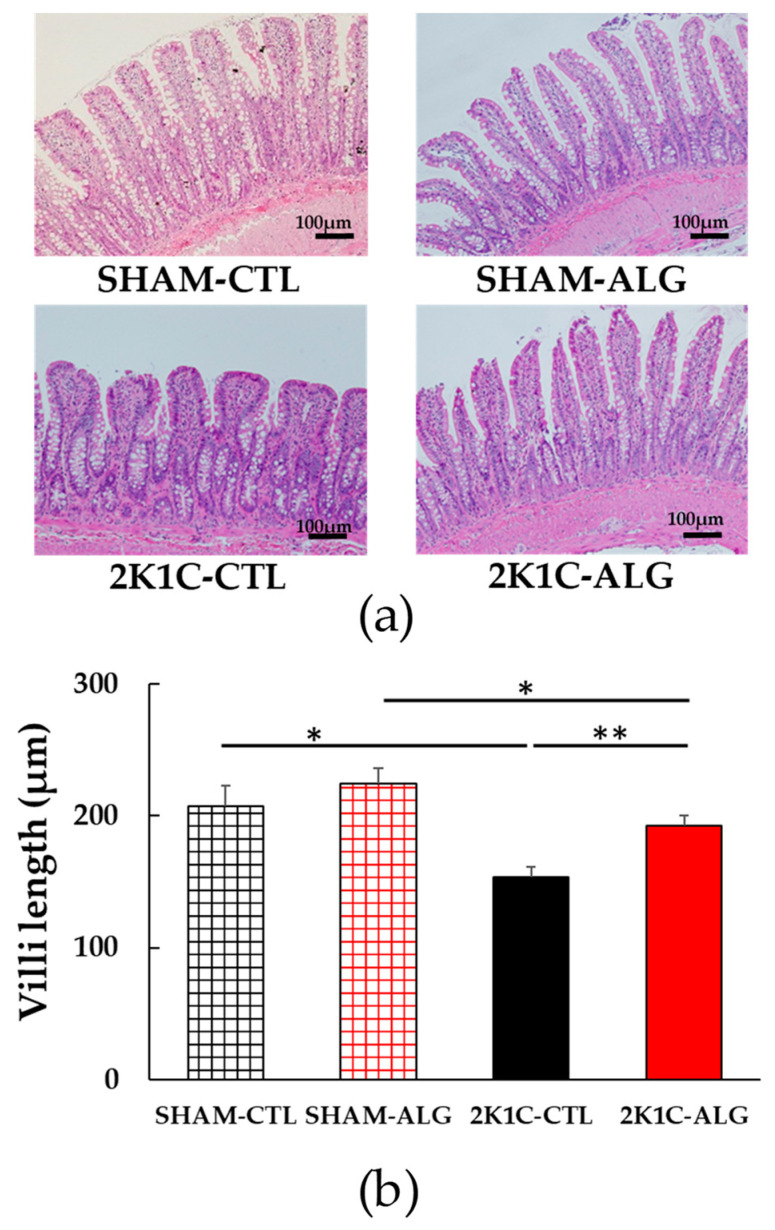
Villi length in the ileum in SHAM or 2K1C rats fed CTL or ALG for 6 weeks. (**a**) Representative micrographs of H&E staining and (**b**) Quantitative villi length of H&E staining. Values are expressed as mean ± SE, *n* = 6. Two-way ANOVA: *p* < 0.05 for animal (SHAM vs. 2K1C) x diet (CTL vs. ALG). * *p* < 0.05 and ** *p* < 0.01. Abbreviations: SHAM, sham-operated control rats; 2K1C, 2-kidney, 1-clip hypertensive rats; CTL, a control diet; ALG, a diet with sodium alginate; SE, standard error; ANOVA, analysis of variance.

The fibrotic area in the ileum was significantly higher in the 2K1C-CTL than in the SHAM-CTL (8.2 ± 1.2 vs. 13.5 ± 1.6%, *p* < 0.05; Figure 3). Compared with 2K1C-CTL, a marginal reduction in fibrotic area was observed in the 2K1C-ALG (10.1 ± 0.8%, *p* = 0.08). However, no significant difference in the fibrotic area was observed between SHAM-CTL and SHAM-ALG (7.9 ± 0.9%). Figure 4 shows a significant elevation in the thickness of the muscle layer compared with those in SHAM-CTL (179.2 ± 6.4 vs. 131.6 ± 8.6 µm, *p* < 0.01). Additionally, the thickness of the muscle layer was significantly lower in the 2K1C-ALG (136.1 ± 11.0 µm) than in the 2K1C-CTL (*p* < 0.01). However, no significant difference in the thickness of the muscle layer was observed between SHAM-CTL and SHAM-ALG (138.6 ± 13.6 µm). As shown in Figure 5, the ratio of goblet cells/villi was found to be higher in the ileum of the 2K1C-CTL group compared to the SHAM-CTL group (22.8 ± 1.2 vs. 30.7 ± 1.3, *p* < 0.01). Compared with 2K1C-CTL, a significant reduction in the ratio of goblet cells/villi was observed in the 2K1C-ALG (29.7 ± 1.9, *p* < 0.01). No significant difference in the ratio of goblet cells/villi was observed between SHAM-CTL and SHAM-ALG (34.6 ± 2.0). The villi length was observed as a significant reduction in 2K1C-CTL compared with those in SHAM-CTL (153.6 ± 7.7 vs. 206.9 ± 16.2 µm, *p* < 0.05; Figure 6). In addition, the villi length was significantly lower in the ileum of the 2K1C-ALG group compared to the SHAM-ALG group (192.1 ± 8.2 vs. 224.2 ± 11.6 µm, *p* < 0.05). A significant increase in villi length was observed in the 2K1C-ALG group than in the 2K1C-CTL group (*p* < 0.01). However, no significant difference in villi length was observed between SHAM-CTL and SHAM-ALG. These findings indicate that dietary sodium alginate intake attenuates the gut pathology deteriorated in the hypertensive animal models, 2K1C rats. In contrast, no differences in gut pathology were found in the ileum of SHAM rats fed a sodium alginate diet compared with those of SHAM rats fed a control diet.

### 2.3. Effects of Dietary Sodium Alginate Intake on Intestinal Tight Junction Proteins in SHAM and 2K1C Rats

**Figure 7:**
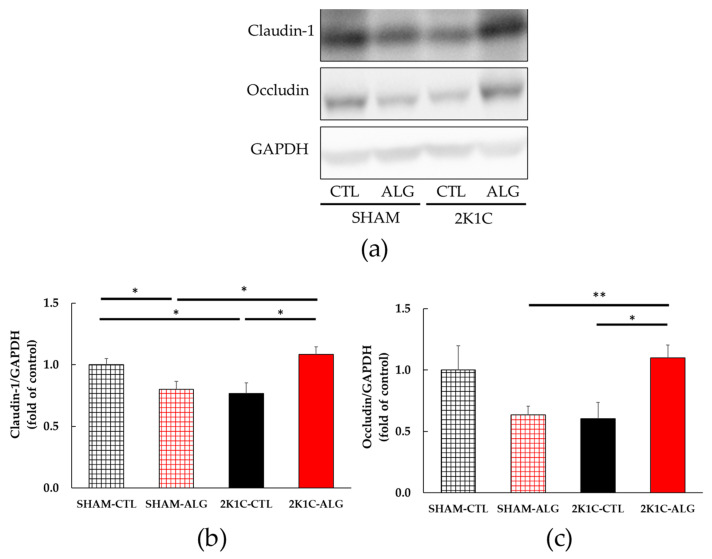
Intestinal tight junction proteins expression in the proximal colon in SHAM or 2K1C rats fed CTL or ALG for 6 weeks. Values are expressed as mean ± SE, *n* = 5–6. (**a**) Western blotting of claudin-1, occludin and GAPDH expression in the proximal colon. (**b**) The relative expressions of claudin-1/GAPDH were quantified. Two-way ANOVA: *p* < 0.01 for animal (SHAM vs. 2K1C) x diet (CTL vs. ALG). (**c**) The relative expressions of occludin/GAPDH were quantified. Two-way ANOVA: *p* < 0.01 for animal x diet. * *p* < 0.05 and ** *p* < 0.01. GAPDH was used as an internal control. Abbreviations: SHAM, sham-operated control rats; 2K1C, 2-kidney, 1-clip hypertensive rats; CTL, a control diet; ALG, a diet with sodium alginate; SE, standard error; ANOVA, analysis of variance.

The results of the two-way analysis of variance indicated a significant interaction between animal models (SHAM or 2K1C rats) and diets (CTL or ALG) on both claudin-1 and occludin, which are intestinal tight junction proteins involved in the gut barrier (Figure 7, animal x diet: *p* < 0.01). The claudin-1 protein expression was significantly lower in the proximal colon of the 2K1C-CTL group than in the SHAM-CTL group (0.77 ± 0.09 vs. 1.00 ± 0.05, *p* < 0.05; Figure 7a). There was a significant elevation in the 2K1C-ALG group (1.08 ± 0.06) compared to the 2K1C-CTL group (*p* < 0.05). A significant increase in the claudin-1 protein level was observed in the 2K1C-ALG group compared to the SHAM-ALG group (0.80 ± 0.07, *p* < 0.05). In addition, the claudin-1 protein expression was significantly lower in the SHAM-ALG group than in the SHAM-CTL (*p* < 0.05). Figure 7b showed that the occludin protein level in 2K1C-ALG was significantly higher compared to that in the SHAM-ALG (1.10 ± 0.11 vs. 0.64 ±0.07, *p* < 0.01), and it was also significantly higher than that in the 2K1C-CTL (0.61 ± 0.13, *p* < 0.05). These studies suggested that sodium alginate intake increased intestinal tight junction proteins in 2K1C rats.

### 2.4. Effects of Dietary Sodium Alginate Intake on Plasma LPS in SHAM and 2K1C Rats

**Figure 8:**
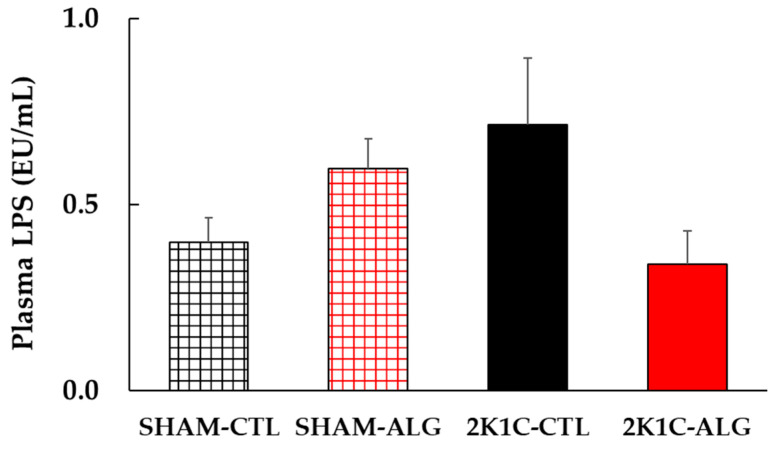
Plasma LPS levels in SHAM or 2K1C rats fed CTL or ALG for 6 weeks. Values are mean ± SE, *n* = 4–6. Two-way ANOVA showed *p* < 0.05 for animal (SHAM vs. 2K1C) x diet (CTL vs. ALG). Abbreviations: SHAM, sham-operated control rats; 2K1C, 2-kidney, 1-clip hypertensive rats; CTL, a control diet; ALG, a diet with sodium alginate; LPS, lipopolysaccharides; SE, standard error; ANOVA, analysis of variance.

The result of the two-way analysis of variance indicated a significant interaction on plasma LPS between animal models (SHAM or 2K1C rats) and diets (CTL or ALG) (Figure 8, animal x diet: *p* < 0.05). The significant interaction indicated that sodium alginate may effect LPS in plasma levels, although a statistically significant difference was detected in the comparison between CTL and ALG (*p* > 0.05) in neither SHAM nor 2K1C in this study.

## 3. Discussion

The present study demonstrated that the BP of 2K1C rats fed a sodium alginate diet decreased compared with that of 2K1C rats fed a control diet (Figure 1 and Figure 2). Previous studies showed that the chronic administration of alginate oligosaccharide attenuated the BP increase in SHR, in high-salt induced hypertensive rats and in Dahl-salt sensitive rats [7,8,9,10,11,13]. These findings indicated that sodium alginate intake may attenuate hypertension in 2K1C rats together with other hypertensive rat models.

In this study, the intake of sodium alginate decreased the thickness of the muscle layer and increased the ratio of goblet cells/villi and villi length in the ileum of the 2K1C rats, but did not significantly decrease the fibrotic area (Figure 3, Figure 4, Figure 5 and Figure 6). Previous studies have reported an increase in the fibrotic area of the gut and the thickness of the muscle layer, as well as a decrease in the ratio of goblet cells/villi and villi length, in hypertensive rat models [14,15,25], which is consistent with the data from this study. Goblet cells primarily protect the host by producing and secreting mucins, which form a mucus layer that isolates microorganisms from the intestinal epithelium, thereby protecting the intestinal barrier [26]. Furthermore, the administration of alginate has been shown to increase goblet cells and mucosal layer integrity in fumonisin B1-induced gut microbial dysbiosis and DSS-induced mice [23,27]. Thus, these findings suggest that sodium alginate intake may improve the gut pathology in 2K1C rats.

Our study showed that the expression of tight junction proteins such as claudin-1 and occludin in the proximal colon was significantly or marginally decreased in 2K1C rats (Figure 7). Previous studies have reported that tight junction proteins in both the ileum and proximal colon were reduced in hypertensive rat models [14,18,19], and that exercise or administration of the angiotensin II type 1 receptor blocker, candesartan, increased these proteins in SHR [18,19]. The tight junction proteins were downregulated in the human colon adenocarcinoma cell line Caco-2 stimulated with LPS [28]. Sodium alginate has been shown to upregulate the expression of tight junction proteins in mice with high-fat diet-induced obesity and cyclophosphamide-induced immunosuppressed BALB/c mice [25,29]. Our findings suggest that sodium alginate intake may impair the gut barrier in 2K1C rats.

LPS is the main component of the outer membrane of Gram-negative bacteria. Plasma LPS is one of the indicators of intestinal permeability. Previous studies have been observed that plasma LPS levels were elevated in SHR and DOCA-salt hypertensive rats [15,17]. Given these studies, the data in the present study suggested that sodium alginate may have a beneficial effect of reducing the increase in plasma LPS levels observed in 2K1C rats (Figure 8). Higher relative abundances of Gram-negative bacteria have been reported to be associated with higher BP in hypertensive patients [30,31]. Additionally, a previous study indicated that the composition and structure of gut microbiota were altered in 2K1C rats, with drastically increasing in relative abundance of the Gram-negative bacteria genera, *Prevotella* [32]. Thus, it is possible that Gram-negative bacteria with LPS on the outer membrane increased in the intestine of 2K1C rats in the present study as well, and that LPS circulated throughout the body as a result of increased intestinal permeability accompanied with increased fibrosis and muscle thickness, and decreased goblet cells and villi length in the gut. Moreover, a study by Wu et al. showed that low doses of LPS infusion (0.3 to 1.2 mg/kg/day) induced systemic inflammation and a long term pressor response, and high doses of infusion (5 to 10 mg/kg/day) induced septic like hypotension [33]. It is suggested that an intraperitoneal injection of a low dose of LPS increases BP [34,35]. An intake of alginate oligosaccharide and polymannuronic acid alleviated blood LPS levels in DSS-induced ulcerative colitis mice, SHR, and C57BL/6J mice fed a high-fat diet and a high-fat and high-sucrose diet [8,12,23,36]. Accordingly, sodium alginate may improve the gut barrier in 2K1C rats, leading to the inhibitory of LPS circulation and then attenuating further BP increase. However, the dysfunction of the intestinal barrier may affect not only circulating LPS, but also other pathways, in the mechanism of decreasing BP. To make it clear, further studies are needed.

Dietary sodium alginate may not achieve BP reduction in normotensive rats, although the mechanism is unclear. One of the possible mechanisms is compensation by intestinal homeostasis. The intestinal modulation, including the intestinal barrier dysfunction, dysbiosis and endotoxemia affects intestinal homeostasis [37]. In a previous study, it was reported that captopril had no effect on the intestine of the Wister Kyoto rats without the changes in gut barrier [14]. Since this study has not tested it, further research is needed to resolve this issue.

In summary, the present study shows that chronic sodium alginate intake for 6 weeks suppressed BP elevation, with a possibly improved gut barrier in 2K1C hypertensive rats. We suppose that the intestinal permeability increase in 2K1C rats may lead to the circulation of LPS or result in the alteration of intestinal homeostasis, either of which could further increase BP. We also suggest that the intake of sodium alginate inhibits the mechanism by gut barrier restoration and alleviates the BP increase in 2K1C rats.

## 4. Materials and Methods

### 4.1. Animals and Treatment

Four-week-old male Sprague Dawley rats were obtained from the Japan SLC Co., Ltd. (Shizuoka, Japan) and were housed in individual cages in a room with a controlled temperature (21 ± 2 °C), humidity (60 ± 10%), and artificial lighting set to a 12 h dark/light cycle. During the preliminary breeding period, the animals had free access to a standard rat chow (CLEA Rodent Diet CE-2, CLEA Japan, Inc., Tokyo, Japan) and tap water. The protocol was approved by the Animal Experiment Committee of Kobe Women’s University (#A276). To establish a 2K1C model of renovascular hypertension, 6-week-old rats were anesthetized with an intraperitoneal injection of a 3-mixed solution (0.2 mL/100 g body weight) of 0.15 mg/kg medetomidine hydrochloride (Nippon Zenyaku Kogyo Co., Ltd., Fukushima, Japan), 2 mg/kg midazolam (Sandoz K.K., Tokyo, Japan), and 2.5 mg/kg butorphanol (Meiji Animal Health Co., Ltd., Kumamoto, Japan). A silver clip with an internal diameter of 0.254 mm was attached to the left renal artery as previously described [2,38,39]. A sham control group (SHAM) of rats received a similar surgical intervention, except that no silver clip was attached. After surgery, both SHAM and 2K1C rats started receiving either a control diet or an experimental diet for 6 weeks.

### 4.2. Experimental Diets

Sodium alginate is purchased from KIMICA Co., Ltd. (Tokyo, Japan). Sodium alginate with a viscosity in the range of 50–80 mPa·s at 20 °C for a 1% aqueous solution was used. The experimental diets were mixed with the sodium alginate and a control diet at a rate of 1:99 (*w*/*w*). The concentration of sodium alginate used in this study was determined based on the antihypertensive effects observed in a previous study that examined the effects of sodium alginate administration [7] and in our study that examined the effects of administration of *Saccharine japonica* which sodium alginate contained approximately 20% of its dry weight [2]. The amount of the sodium alginate given to rats in this experiment is equivalent to 15–60 g per day in a human weighing 60 kg and consuming 1.5 kg of food daily, assuming that a rat in this experiment weighs 250 g and consumes 25 g of food daily. In this experiment, a standard diet (CLEA Rodent Diet CE-2, CLEA Japan, Inc., Tokyo, Japan) was used as the control diet. The experiments were also performed adjusting the energy intake to be equal among the groups.

### 4.3. Protocol Observing the Effect of Sodium Alginate Intake on BP, Gut Morphology, Intestinal Tight Junction Proteins, and Plasma LPS Levels

After surgery, the rats received either a control diet (CTL) or a diet with 1.0% (*w*/*w*) sodium alginate (ALG) for 6 weeks. The rats were divided into the following groups: SHAM-CTL, SHAM-ALG, 2K1C-CTL, and 2K1C-ALG (*n* = 6 for each group). The SBP and body weight were measured weekly as described below. Body weight did not significantly change among the groups throughout the experiments. At the end of the experiments, the mean arterial BP (MAP) was measured under anesthesia using the same mixed anesthetic agents as used during surgery as described above. After the measurement of MAP, blood samples were collected under deep anesthesia using the same anesthetic agents; thereafter, rats were euthanized. The blood samples were centrifuged at 3000× *g* for 10 min at 4 °C to collect plasma for LPS assay. The ileum and proximal colon were isolated for pathological analysis and Western blotting, respectively. The resulting plasma and proximal colon were frozen at −80 °C.

### 4.4. Blood Pressure Measurements

SBP was evaluated as described previously [2,38,39] and measured using a tail-cuff method with an MK-1030 NIBP monitor (Muromachi Kikai Co., Ltd., Tokyo, Japan) in conscious animals each week. Before the measurements, the rats were restrained and placed in a chamber at 38 °C and maintained for 10 min to readily detect the arterial pulsation in the tail and stabilize their SBP. SBP was measured 10 times consecutively, and the average value was used for evaluation.

For the MAP measurement, the left femoral artery was catheterized using polyethylene tubing (PE-10; Becton Dickinson, Sparks, MD, USA) attached to a pressure transducer and recorder, as previously described [2,38,39]. The MAP was continuously monitored using a PowerLab System with a signal amplifier (AD Instruments, Belle Vista, Australia) attached to a BP transducer (AR611G; Nihon Kohden Corp., Tokyo, Japan). The data were collected during the last 3 min of a 10 min stabilization period.

### 4.5. Measurement of Plasma LPS Concentration

Plasma LPS concentrations were measured by the Pierce^TM^ Chromogenic Endotoxin Quant Kit (Thermo Fisher Scientific Co., Ltd., Waltham, MA, USA). Briefly, plasma samples were incubated at 70 °C for 15 min. Sterile and endotoxin-free materials were used for all the experiments to guarantee samples and test integrity.

### 4.6. Histological Analysis

The ileum was collected from all animals and fixed in 10% formalin. The samples were then processed for paraffin embedding and sectioning. Paraffin sections were stained with hematoxylin and eosin (H&E) and Masson’s trichrome to visualize the general morphology and fibrosis. The glass slides were then examined using an Olympus microscope (Olympus Co., Tokyo, Japan). The degree of fibrosis, thickness of the smooth muscle cell layer, and the ratio of goblet cells to villi and villi length were quantified using the ImageJ software (National Institute of Health, Bethesda, MD, USA).

### 4.7. Western Blotting

The proximal colon was homogenized in ice-cold Pierce RIPA Lysis and Extraction Buffer^®^ with Halt™ Protease and Phosphatase Inhibitor Cocktail (Thermo Fisher Scientific, Waltham, MA, USA). The total protein (50 µg) concentration was determined using the BCA protein assay kit (Thermo Fisher Scientific, Waltham, MA, USA). After separation on 10% sodium dodecyl sulfate (SDS)–polyacrylamide gel electrophoresis gels (Bio-rad Laboratories, Inc., Hercules, CA, USA), the proteins were transferred to nitrocellulose membranes (Biorad) and then blocked with 5% (*w*/*v*) non-fat dry milk in Tris-buffered saline that contained 0.1% Tween 20 for 1 h at room temperature. The membranes were then incubated with the indicated antibodies, including rabbit anti-GAPDH (1:1000, Cell Signaling Technology, Beverly, MA, USA; #5174), mouse anti-claudin-1 (1:1000, Santa Cruz Biotechnology, TX, USA; #sc-166338), and mouse anti-occludin (1:1000, Santa Cruz Biotechnology; #sc-133256). The primary antibodies were incubated at 4 °C overnight and followed by the incubations of the horseradish peroxidase conjugated anti-rabbit secondary antibodies (1:10,000, Jackson Immuno Research Laboratories, West Grove, PA, USA; #711-035-152) or anti-mouse secondary antibodies (1:10,000, Jackson Immuno Research Laboratories; #115-005-003) for 1 h at room temperature. Immunoreactivity was detected using the Clarity Western ECL Substrate (Bio-Rad) and the cooled CCD camera system Lumino Graph I (ATTO Co., Ltd., Tokyo, Japan). Densitometry of the protein bands was performed using the ImageJ software (National Institute of Health, Bethesda, MD, USA).

### 4.8. Statistical Analysis

Data are expressed as the mean ± standard error (SE). The results were evaluated using an analysis of variance (ANOVA). The statistical analyses were conducted using the Statistical Package for the Social Sciences version 23 (IBM Co., Chicago, IL, USA). A *p* value of <0.05 was considered statistically significant.

## 5. Conclusions

This study indicated that sodium alginate intake may attenuate hypertension in 2K1C rats by improving the gut barrier. Sodium alginate may help prevent renovascular hypertension.

## Data Availability

Not applicable.
